# Bilateral macronodular adrenocortical disease: a single centre experience

**DOI:** 10.1530/EC-24-0664

**Published:** 2025-02-17

**Authors:** Anuj Ban, Rohit Barnabas, Manjiri Karlekar, Anurag Ranjan Lila, Chethan Yami Channaiah, Saba Samad Memon, Virendra A Patil, Vijaya Sarathi, Gwendolyn Fernandes, Hemangini Thakkar, Sameer Rege, Nalini S Shah, Tushar Bandgar

**Affiliations:** ^1^ Department of Endocrinology and Metabolism, Seth G S Medical College and KEM Hospital, Mumbai, India; ^2^ Department of Endocrinology, Vydehi Institute of Medical Sciences and Research Centre, Bangalore, India; ^3^ Department of Pathology, Seth GSMC and KEMH, Mumbai, India; ^4^ Department of Radiology, Seth GSMC and KEMH, Mumbai, India; ^5^ Department of General Surgery, Seth GSMC and KEMH, Mumbai, India

**Keywords:** BMAD, Cushing’s syndrome, CT scan, AIMAH, adrenal Cushing’s, PBMAH

## Abstract

**Background:**

Data on bilateral macronodular adrenocortical disease (BMAD) with respect to computed tomography (CT) scan characteristics (attenuation and washout) and long-term follow-up are limited. This study aims to describe BMAD patients managed in a single centre.

**Methods:**

BMAD was defined by the presence of bilateral adrenal macronodules (>1 cm) on CT. Clinical, biochemical, radiological, genetic characteristics, management and follow-up of 22 BMAD patients were studied retrospectively.

**Results:**

The median age (range) at presentation was 49.5 (23–83) years, predominantly observed in females (16/22). Eighteen (82%) patients were incidentally diagnosed (11 with mild autonomous cortisol secretion (MACS) and seven non-secretory), three (13.7%) presented with overt Cushing’s syndrome (CS), and one (4.5%) had androgen excess (without CS features). On CT, the dominant nodule’s median (range) size was 2.6(1.6–9.5) cm. 77.8% (14/18) of adrenal nodules were lipid-rich, and 93.3% (14/15) of the nodules exhibited good washout. Genetic analysis was available for eight patients; one had a novel germline *ARMC5* variant, and two had *MEN-1* gene mutations. Three overt CS and one androgen-secreting patient underwent total bilateral adrenalectomy; histopathology showed macronodular hyperplasia with internodular hypertrophy. Only one (1/8) patient from the MACS group developed a new comorbidity (diabetes mellitus) after a median follow-up of 6.4 (0.5–12.4) years, while none of the non-secretory group patients developed new comorbidities after a median follow-up of 1.4 (0.8–12.2) years.

**Conclusion:**

Most BMAD patients presented without overt hormonal excess, and none developed overt CS on follow-up. Detailed CT characteristics of BMAD nodules may help in radiological diagnosis in bilateral adrenal incidentalomas.

## Introduction

Bilateral macronodular adrenocortical disease (BMAD) is often an incidental radiological finding, typically in the fifth and sixth decades of life (1). Most BMAD series report that patients have either mild autonomous cortisol secretion (MACS) or overt Cushing’s syndrome (2, 3). Amongst the cohort of endogenous Cushing’s syndrome (CS), BMAD accounts for only <2–6% of cases (4). BMAD is characterised by adrenal macronodules (>1.0 cm) and variable levels of cortisol excess, and there are no clearly established clinical diagnostic criteria. A recent expert review suggests that radiologically diagnosed cases of BMAD may have normocortisolism at presentation (apparently non-secretory) and need annual follow-up with 1 mg dexamethasone suppression test (5). BMAD may rarely be associated with genetic syndromes such as multiple endocrine neoplasia type 1 (MEN1), familial adenomatous polyposis and hereditary leiomyomatosis and renal cell cancer (6, 7, 8, 9). Other germline mutations causing isolated BMAD include *ARMC5* and *KDM1A* (10, 11). A confirmatory diagnosis of BMAD is typically made through histopathology; however, many patients are managed medically and are often diagnosed based on a combination of clinical, biochemical and radiological (computed tomography (CT) scan) features, along with the exclusion of other causes of bilateral adrenal enlargement. Though adrenal gland volume and its correlation with biochemical severity is described in a series of BMAD patients (12), data regarding CT scan characteristics, such as attenuation values and washout characteristics specific to BMAD, is limited (13). Data on functional imaging, such as ^18^F-FDG PET, and its utility in distinguishing BMAD from other bilateral adrenal lesions due to infiltrative or infective disorders, is also scarce. Patients with overt CS typically require unilateral or bilateral adrenalectomy, while there is limited follow-up data available for patients with MACS who are conservatively managed (14). This study aims to describe the clinical, biochemical, radiological, genetic characteristics, management and follow-up of BMAD patients managed at a single centre.

## Patients and methods

This retrospective study was approved by the Institutional Review Board (Institutional Ethics Committee of Seth GS Medical College and KEM Hospital, Mumbai, India), and a waiver of consent was obtained (EC/OA-17/2023). The records of patients with BMAD who had presented to our institution between 2010 and 2023 were reviewed. Information retrieved from the records included baseline clinical features, hormonal profile, radiology, histopathologic findings for those who underwent bilateral adrenalectomy, genetics and follow-up data (when available for more than 6 months from diagnosis).

### Diagnosis

Patients with clinically evident, ACTH-independent endogenous CS with bilateral adrenal macronodules (>1 cm) on imaging were diagnosed as BMAD with overt CS. For patients referred for incidental bilateral adrenal macronodules, an overnight dexamethasone suppression test (ONDST) was performed. MACS was defined as serum cortisol >1.8 μg/dL after an ONDST without specific clinical signs of CS. Patients having ONDST cortisol ≤1.8 μg/dL were classified as non-secretory BMAD after excluding other causes of bilateral adrenal enlargement (such as infiltrative/infective/neoplastic aetiologies). Tests for aldosterone excess, catecholamines, dehydroepiandrosterone sulfate (DHEAS) and testosterone were performed in selected patients. The diagnosis of BMAD was confirmed on histopathology in operated cases.

### Assays

Serum cortisol was measured by a solid-phase competitive chemiluminescent enzyme immunoassay (CLIA) (Siemens Healthcare, Germany) with an analytical sensitivity of 0.2 μg/dL. The cortisol assay’s intra- and inter-assay coefficients of variability (CV) were 6.9 and 7.3%, respectively. Adrenocorticotropic hormone was measured by a solid-phase, two-site sequential chemiluminescence assay on Liaison (Diasorin, Italy). The intra- and inter-assay CV were 4.9 and 8.9%, respectively. Plasma free metanephrines and normetanephrines were measured by ELISA. All other hormonal investigations were done by CLIA (Liaison, Diasorin, Italy). Intra-assay and inter-assay coefficients of variation were less than 8 and 10%, respectively, for the estimation of all hormones.

### Radiology

Imaging of the abdomen was performed on a 64-slice multidetector CT system (Brilliance 64, Philips Healthcare, Netherlands) using a standardised protocol as described earlier in detail (15). Imaging characteristics such as size, CT attenuation and washout pattern were noted for the largest nodule in each patient’s adrenal glands. Nodules were labelled lipid-rich if the unenhanced CT attenuation value was ≤10 Hounsfield unit (HU). Absolute and relative washout were calculated using standard formulae (with unenhanced, +60 s and +15 min attenuation values). Good washout was defined by relative washout ≥40% (16).

### Genotype analysis

Genomic DNA was extracted from peripheral blood leukocytes using standard techniques. Clinical exome sequencing comprising 6700 genes was available for eight patients with BMAD. The libraries were sequenced to a mean >80–100× coverage on the Illumina sequencing platform. The sequences obtained were aligned to the human reference genome (GRCh38.p13) using the Sentieon aligner. The Genome Analysis Toolkit (GATK) best-practices framework was followed to identify variants in the sample using the Sentieon (v201808.07). Genes (*ARMC5*, *KDM1A*, *PDE8B*, *PDE11A*, *MEN1*, *PRKACA*, *APC* and *FH*) implicated in the pathogenesis of BMAD were analysed. The functional implication of variants was predicted using *in silico* tools (MutationTaster2, Polymorphism Phenotyping v2, Sorting Intolerant from Tolerant and NetGene2). The variants’ minor allele frequency was checked in databases like 1000 genomes and gnomAD.

### Statistical analysis

Qualitative data were represented in the form of frequency and percentage. The association between qualitative variables was assessed by the χ2 test with continuity correction or Fisher’s exact test, as appropriate. Quantitative data were represented using mean ± standard deviation or median and interquartile range, wherever appropriate. Quantitative data analysis between two groups was done using the unpaired *t*-test/Mann-Whitney test, while a one-way ANOVA test was used for comparing more than two groups. For all statistical tests, *P* = <0.05 was considered significant. Statistical analysis was performed using SPSS Statistics, version 26.0 (IBM, USA).

## Results

The median age (range) at presentation was 49.5 (23–83) years, the majority being female (16/22, 72.7%). Out of the 22 patients, three (13.7%) presented with overt CS, one (4.5%) had hirsutism and infertility (without CS features), 11 (50%) presented with incidental adrenal masses and had MACS, and seven (31.9%) were non-secretory BMAD. Demographic, clinical, hormonal and radiological characteristics of these sub-cohorts are mentioned in Table 1, and per-patient details are added in supplemental data (see section on Supplementary materials given at the end of the article). The prevalence of hypertension (HTN) in patients with overt CS and MACS was 100% (3/3) and 54.5% (6/11), while that of diabetes mellitus (DM) was 66.7% (2/3) and 27.3% (3/11), respectively. None of the seven non-secretory BMAD patients had HTN or DM. The serum 8 AM cortisol, ONDST cortisol and ACTH levels in various sub-cohorts are detailed in Table 1. One 28-year-old female patient (P5) with pure androgen-secreting BMAD had a normal serum ONDST cortisol (0.67 μg/dL), along with elevated serum androgens, DHEAS (915.4 μg/dL, normal range 95.8–511 μg/dL) and testosterone (2.7 ng/mL), with serum gonadotropin levels of serum FSH (follicle-stimulating hormone) and LH (luteinizing hormone) being 7.5 and 10.2 mIU/mL, respectively. Basal 08.00 AM serum LCMS/MS steroid profile was available for three patients, one overt CS with *ARMC5* mutation (P9) and two MACS patients (P10 and P12); none of them had elevated steroid precursors (supplementary data). Testing results for abnormal adrenal receptors (upright posture, mixed meal, metoclopramide, gonadotropin-releasing hormone, vasopressin and glucagon) were available for P7 having MACS. A 206% rise in serum cortisol (6.6–20.2 μg/dL) was seen after 30 min of vasopressin injection, while other stimuli did not increase cortisol levels.

**Table 1:** Demographic, clinical, biochemical and treatment details of BMAD across groups based on secretory status.

	Overt CS (*n* = 3)	MACS (*n* = 11)	Androgen secretory (*n* = 1)	Non-secretory (*n* = 7)
Age at diagnosis (years) median (range)	47 (30.65)	51 (23.72)	28	50 (38.83)
Gender (M:F)	1:2	2:9	0:1	3:4
Hypertension	3/3 (100%)	6/11 (54.5%)	0/1	0/7 (0%)
Diabetes mellitus	2/3 (66.7%)	3/11 (27.3%)	0/1	0/7 (0%)
Biochemistry (median (range))				
Basal cortisol (μg/dL)	26 (11.3, 46.6)	18.6 (5.7, 36.3)	7.3	11.1 (6.8, 14.4)
ONDST cortisol (μg/dL)	34 (8.5, 39.9)	3.7 (1.9, 12.3)	0.67	1.2 (0.9, 1.5)
Basal ACTH (pg/mL)	5.1 (3.9, 5.8)	12.4 (7.6, 31.6)	12.3	19 (9.4, 118)
DHEAS (μg/dL)	28.6 (28.2, 29) *n* = 2	51.7 (15, 121) *n* = 6	915.4	26.5 (10.7, 91.2) *n* = 3
Radiology				
Nodule size (cm) median (range)	3.3 (2.8, 4.7), *n* = 3	2.6 (1.6, 5.2), *n* = 11	9.5	2.2 (1.7, 4.0), *n* = 7
Basal CT attenuation (HU) median (range)	−3.5 (−6.8, 0.5), *n* = 2	7 (−8, 25), *n* = 9	41	−1 (−11, 40), *n* = 6
Lipid rich	2 (100%), *n* = 2	7 (77.8%), *n* = 9	0/1	5 (83.3%), *n* = 6
Good washout	2 (100%), *n* = 2	8 (100%), *n* = 8	1/1	3 (75%), *n* = 4
Treatment	TBA – 3/3 (1 awaiting)	Observation – 11/11	TBA	Observation – 7/7
		Medical therapy – 1/11 on follow-up		
Genetics (*n* = 8)	1 *ARMC5*, *n* = 2	1 *MEN-1*, *n* = 4	Negative	1 *MEN-1*, *n* = 1
Follow-up duration (years) median (range)	0.66, 5.66 *n* = 2	6.4 (0.5, 12.4) *n* = 8	6	1.4 (0.8, 12.2) *n* = 6

Abbreviations: ACTH, adrenocorticotropic hormone; CS, Cushing’s syndrome; CT, computed tomography; DHEAS, dehydroepiandrosterone sulfate; MACS, mild autonomous cortisol secretion; ONDST, overnight dexamethasone suppression test; TBA, total bilateral adrenalectomy; HU, Hounsfield unit.

**Figure 1:**
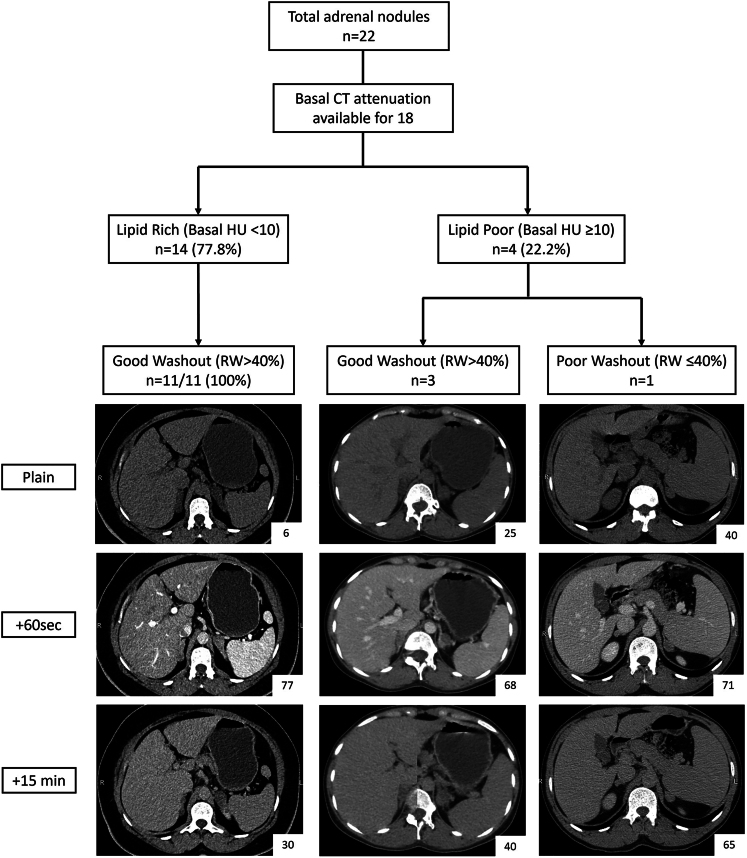
Radiological characterisation of BMAD nodules. CT HUs of dominant nodules are mentioned in the right lower corner box.

CT characteristics of adrenal lesions are described in Table 2 and Fig. 1. The dominant adrenal nodule in each patient was assessed for basal CT attenuation and washout characteristics. The dominant nodule’s median (range) size was 2.6 (1.6–9.5) cm. The median size of the dominant nodule was largest for the androgen-secreting BMAD (9.5 cm), followed by the overt CS group (3.3 cm), MACS (2.6 cm) and non-secretory groups (2.2 cm), although these differences were not statistically significant. A total of 77.8% (14/18) of adrenal nodules were lipid-rich (basal HU <10) and 93.3% (14/15) exhibited good washout (relative washout >40%), which is consistent with benign lesions. The four patients with lipid-poor (basal HU ≥10) adrenal lesions belonged to the androgen-secreting BMAD (*n* = 1, 41 HU), MACS (*n* = 2, 12 and 25 HU) and non-secretory BMAD group (*n* = 1, 40 HU). The patients with lipid-poor adrenal lesions had good washout characteristics (*n* = 3), except for one with non-secretory BMAD (relative washout: 8.5%, P20). Based on such radiological characteristics, P20 was initially thought to have infiltrative (metastasis/infection) lesions. He was normotensive and biochemically had normal adrenal cortical and medullary function. An ^18^F-FDG PET scan did not reveal any primary malignancy or infectious foci elsewhere, and a repeat adrenal CT scan after 1.5 years of the initial presentation showed similar characteristics as the baseline scan. ^18^F-FDG PET was available for three patients, one with overt CS (P14) and two with non-secretory BMAD (P18 and P20), with the adrenal nodule SUVmax values being 5.8, 2.0 and 6.2, respectively.

**Table 2:** Detailed radiological findings of BMAD patients.

Patient	Function	Dominant side, largest dimension (cm)	Basal, arterial, venous, delayed HU	AW (%), RW (%)	Lipid rich/poor	Washout good/poor	Contralateral nodule size (cm)	Contralateral nodule character
3	S–SC	Right, 5.2	8, -, 36, 17	68, 53	Rich	Good	2.8	Concordant
4	S–C	Right, 3.3	−6, 20, 34, 10	60, 71	Rich	Good	2.5	Concordant
5	S–androgen	Right, 9.5	41, 155, 124, 70	65, 44	Poor	Good	2.7	Concordant
6	S–SC	Left, 1.6	9, 72, 82, 42	55, 49	Rich	Good	1.1	Concordant
8	S–SC	Left, 2.6	−7, 15, 35, 4	74, 89	Rich	Good	1.3	Concordant
9	S–C	Left, 4.7	−1, 14, 35, 8	75, 77	Rich	Good	4.3	Concordant
10	S–SC	Left, 2.3	6, 26, 77, 30	66, 61	Rich	Good	1.7	Concordant
11	S–SC	Right, 2.6	−5, 7, 28, 16	36, 43	Rich	Good	1.7	Concordant
12	S- SC	Left, 2.1	12, 116, 92, 30	78, 67	Poor	Good	1.5	Concordant
13	S–SC	Left, 2.6	25, 37, 68, 40	65, 41	Poor	Good	1.8	Concordant
15	S–SC	Right, 3.1	−8, 9, 44, 13	60, 70	Rich	Good	1.2	Concordant
16	NS	Left, 2	3, 69, 62, 11	86, 82	Rich	Good	2	Concordant
18	NS	Left, 2.3	−11, 35, 57, 13	65, 77	Rich	Good	1.3	Concordant
20	NS	Left, 4	40, 54, 71, 65	19, 8	Poor	Poor	2	Concordant
21	NS	Left, 1.7	−10, 5, 20, 6	47, 70	Rich	Good	1.3	Concordant

Abbreviations: AW, absolute washout; C, overt Cushing’s; HU, Hounsfield unit; NS, non-secretory; RW, relative washout; S, secretory; SC, subclinical Cushing’s.

Clinical exome sequencing was available for eight patients (two overt CS, four MACS, one androgen-secreting and one non-secretory), of whom one patient with overt CS had a novel *ARMC5* germline variant (Exon 3, c.798C>A, p.Ser266Arg). The p.Ser266Arg variant has not been reported in the 1000 genomes, gnomAD (v3.1), gnomAD (v2) or topmed. The *in silico* predictions of the variant are probably damaging by PolyPhen-2 (HumDiv) and damaging by SIFT and LRT. The reference codon is conserved across species. Pathogenic *MEN-1* mutations were seen in two patients, one with MACS (c.1012del) and the other with non-secretory BMAD (Exon 2–10 deletion). These patients also had other components of MEN-1 syndrome, such as primary hyperparathyroidism and pancreatic neuroendocrine tumours, and BMAD was diagnosed incidentally during screening. All three patients with germline variants were apparently sporadic and did not have any family history of a similar illness.

**Figure 2:**
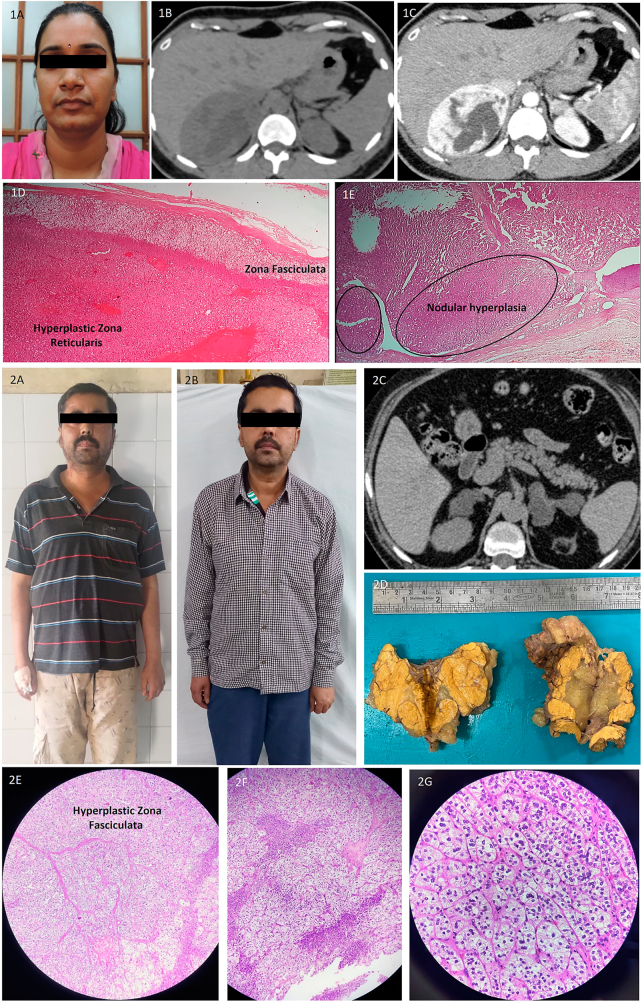
Androgen-secreting (P5) – (1A) clinical picture showing hyperandrogenic features, (1B, C) bilateral adrenal masses showing intense heterogeneous contrast enhancement, (1D) scanner view showing hyperplastic adrenal cortex with macronodules involving zona reticularis (Hematoxylin and Eosin (H&E) ×50), (1E) low power magnification showing macronodules of varying sizes (H&E ×100) ARMC5+ patient (P9) – (2A) clinical picture showing cushingoid features, (2B) resolution of cushingoid appearance post total bilateral adrenalectomy, (2C) CT showing multiple bilateral lipid-rich nodules with significantly enlarged adrenals, (2D) gross pathology showing bilateral grossly enlarged adrenal glands with multiple nodules, (2E–G): nodules of varying sizes composed of polyhedral cells with clear, vacuolated cytoplasm and small, bland nuclei. (2E/F – H&E ×100, 2G – H&E ×400).

Two overt CS patients are in remission after total bilateral adrenalectomy (TBA), while one patient is awaiting surgery. The patient with androgen-secreting BMAD (P5) has normal serum androgen levels (DHEAS: 137 μg/dL), testosterone: 0.15 ng/mL) after TBA. On pathological examination, the weight of the adrenal glands varied from 10 to 110 g. Macronodular hyperplasia was observed, with nodule sizes varying from 0.7 to 7 cm and hyperplastic internodular tissue. The patient with androgen-secreting BMAD (P5) had hyperplastic zona reticularis, and two patients with overt CS (one with *ARMC5* mutation (P9)) were reported to have hyperplastic zona fasciculata. Figure 2 illustrates clinical, radiological and histopathological details of patients P5 and P9.

Eight patients with MACS (8/11) were followed up for a median (range) duration of 6.4 (0.5–12.4) years. None of these patients developed overt CS or new comorbidities (HTN, DM, acute coronary syndrome, stroke and fractures) except one who developed DM, after which she was recently initiated on oral ketoconazole (400 mg once daily, 18:00 h), and her glycaemic control is better without oral anti-diabetic agents. In addition, the median cortisol after ONDST at the last follow-up was available for six out of eight MACS patients and was similar to the baseline evaluation (3.7 vs 3.3). Patients with non-secretory BMAD were also followed up for a median (range) duration of 1.4 (0.8–12.2) years, and none developed any new comorbidities by their last follow-up. Among the non-secretory BMAD group, ONDST cortisol on follow-up (12 years) was available in one patient and remained suppressed.

## Discussion

Our study cohort of BMAD patients is the first series from India and provides valuable insights into disease heterogeneity. The novel feature of our study is the detailed CT characteristics, reporting that most BMAD nodules are lipid-rich (78%) and have good relative washout (93%). This observation can help delineate BMAD from bilateral adrenal masses with lipid-poor/poor washout characteristics. We also report the first case of a premenopausal female with pure androgen-secreting BMAD.

The presentation of BMAD at our centre is similar to that reported worldwide: female predominance, usual presentation in the fifth-sixth decade, a larger proportion of BMAD being MACS/non-secretory (82%) and BMAD constituting ∼2% (14/703) of endogenous CS patients (1, 2, 3, 4). However, the overall number of BMAD patients, despite the inclusion of non-secretory cases managed at our centre is relatively small. This could be due to underdiagnosis due to a lack of awareness about this rare condition among radiologists or reflects an ethnic variation in disease prevalence. Metabolic syndrome prevalence in our cohort is proportional to the cortisol excess or severity at presentation, with the highest among overt CS (100% HTN, 67% DM), followed by MACS (54.5% HTN, 25.3% DM) and the non-secretory group (0% HTN or DM). Also, all our patients with overt CS had suppressed ACTH at baseline. In contrast, patients with MACS had a trend of relatively lower ACTH values as compared to non-secretory BMAD patients (12.4 vs 19.0 pg/mL *P* = 0.09), which is due to incomplete suppression of the hypothalamic-pituitary-adrenal axis by the modest cortisol excess.

As per the latest consensus, overnight dexamethasone-suppressed serum cortisol >1.8 μg/dL defines cortisol excess, and the presence or absence of clinical CS classifies it as MACS or overt CS (17). The cortisol response to varied stimuli (glucose-dependent insulinotropic polypeptide, vasopressin, β-adrenergic agonist, LH/hCG, serotonin, octreotide and glucagon) is reported in about 87% (28/32) of BMAD patients, with the most common being vasopressin response (18). We have done such detailed testing for only one of the earliest patients of BMAD with MACS and found a positive cortisol response to vasopressin. As per the expert review, testing with specific stimuli such as mixed meal, upright posture and GnRH is suggested in patients with overt CS, as it may have implications in medical management and guidance for genetic testing (5). *ARMC5* is the most common germline mutation seen exclusively with cortisol-excess BMAD (19). Genetic yield is further higher in cases with severe hypercortisolism and larger adrenals (3). None of our patients with MACS or non-secretory BMAD had an *ARMC5* mutation, and one patient with overt CS harboured a novel *ARMC5* variant. Meningioma has been reported in patients with *ARMC5* mutation (20); it was not seen in our patient. *MEN1* mutations were seen in two BMAD patients, one with MACS and the other non-secretory. The prevalence of BMAD (2.3%, 2/86) in our centre’s MEN1 cohort is similar to a previous study (21) (1.3%, 9/715).

Management of BMAD patients varies widely from observation to medical management and TBA, depending on the severity at presentation (5). In our cohort, patients with overt CS underwent TBA, and no patient was subjected to unilateral adrenal surgery. All three patients who underwent TBA exhibited severe clinical and biochemical features. Two female patients were planning to conceive in the near future, while the third patient, a male, carried a germline ARMC5 variant. Hence, TBA was performed to ensure long-term remission. Unilateral adrenalectomy is also suggested as an alternative surgical treatment approach in select cases, though recent data have reported high biochemical recurrence rates and even higher mortality (22, 23). Those with MACS and non-secretory BMAD were observed and followed up with clinical parameters and biochemistry. Literature regarding such patients’ morbidity, mortality and outcome is scarce. Notably, none of these patients developed overt CS after a median follow-up of 5 years, and only one patient developed a new onset of DM. Other comorbidities such as ACS, stroke and fractures were not apparent. However, bone health was not studied with Dual X-ray absorptiometry at baseline or follow-up.

BMAD on imaging is characterised by bilateral enlargement of adrenals with one or several macronodules (largest diameter >10 mm) in both glands. Volumetric assessment of adrenal glands and its correlation with biochemical severity has been described (12). Only a few case reports and a single case series have described CT attenuation values without mentioning washout characteristics (supplemental data) (13). In our cohort, we found that 73.2% (14/18) of adrenal nodules were lipid-rich, and 93.3% (14/15) exhibited good washout characteristics. This observation can help delineate BMAD from bilateral adrenal masses such as metastasis/infiltration/infection/neoplasm, which are reported to have lipid-poor/poor washout characteristics in the majority (24). Of note, pheochromocytoma can be ruled out with confidence if the lesion is lipid-rich, but in the case of a lipid-poor good washout lesion, it remains a differential, as ∼30% of pheochromocytomas have such characteristics (25). These findings are important as most BMAD are incidentally discovered on CT scan imaging, and radiology-based guidance to probable aetiology will help the clinician to better triage the further workup. Larger studies are required to validate or refine our findings further.

To date, androgen and cortisol co-secretion has been described in three BMAD patients (26, 27, 28), and pure androgen secretion has been documented in a male patient having BMAD (29). The androgen excess in this male patient was clinically silent, and the serum steroid profile gave a diagnostic clue, as it showed high DHEA, DHEAS and 17-hydroxypregnenolone levels. The case also highlights the role of a detailed steroid profile, which may help diagnose such rare BMAD variants. A detailed LCMS/MS steroid profile was available for a few recent patients managed at our centre, though it did not suggest any androgen excess or accumulation of steroid precursors in them. We describe a 28-year-old female patient in our cohort of BMAD with pure androgen secretion (P5), presenting with hirsutism and oligomenorrhoea, who underwent TBA, leading to clinical and biochemical remission. Our patient’s imaging and histopathological characteristics were similar to the previously reported patient. They demonstrated bilateral macronodular hyperplasia of zona reticularis, confirmed by immunoblots showing positive staining for enzymes in the steroidogenic pathway involved in adrenal androgen production. Similarly, in our patient, the histopathology showed hyperplastic zona reticularis, though studies of steroidogenic enzyme activity and mRNA expression are unavailable. Interestingly, both these patients showed large adrenals on CT imaging with heterogeneous and intense contrast enhancement, which may raise a suspicion of bilateral pheochromocytoma.

The main limitation of this study was its retrospective design with inherent drawbacks. Genetic analysis was available for a limited number of patients, and somatic mutations were not studied in patients who underwent TBA. Bone health assessment via DXA scan was lacking in our study. Detailed biochemical investigations and radiology to assess adrenal nodular growth were not available on follow-up.

## Conclusion

In our study, most BMAD patients presented without overt hormonal excess and could be managed medically or through observation, with none developing overt CS on follow-up. This study provides detailed CT characteristics of BMAD nodules, including washout parameters, which may help in radiological diagnosis in patients with bilateral adrenal incidentalomas.

## Supplementary materials



## Declaration of interest

The authors declare that there is no conflict of interest that could be perceived as prejudicing the impartiality of the work.

## Funding

This work did not receive any specific grant from any funding agency in the public, commercial or not-for-profit sector.

## Ethics approval

This study was conducted after approval by the Institutional Ethics Committee of Seth GS Medical College and KEM Hospital (EC/OA-17/2023), Mumbai. Individual patient identity was kept confidential and coded prior to analysis.
