# Supplementary material for: Bilateral macronodular adrenocortical disease: a single centre experience
Source: Endocr Connect. 2025 Feb 17;14(3):e240664. doi: 10.1530/EC-24-0664 (PMC11850045; doi:10.1530/EC-24-0664)
Supplement: Supplementary file 1 [file supplementary_materials.pdf]

Supplementary Data

Supplementary Table 1 Secretory Group – Clinical, biochemical, genetics, treatment and outcome details

	Patient 1	Patient 2	Patient 3	Patient 4	Patient 5	Patient 6	Patient 7	Patient 8	Patient 9	Patient 10	Patient 11	Patient 12	Patient 13	Patient 14	Patient 15
Age yrs	36	20	23	48	28	63	64	49	47	51	62	41	23	65	72
Sex	Female	Female	Female	Female	Female	Female	Male	Female	Male	Female	Male	Female	Female	Female	Female
Presentation 1-overt CS;2-hirsutism, 3-Incidental, 4-DM, 5- HTN	3	3, 5	3	1,5	2	3	3, 5	2, 3, 5	1,4,5	3,4,5	3,4,5	3	3	1,4,5	3,4,5
Family history	-ve	-ve	-ve	-ve	-ve	-ve	-ve	-ve	-ve	-ve	-ve	-ve	-ve	-ve	-ve
Basal cortisol mcg/dl	22.23	10.3	36.3	26	7.3	22.43	28.3	14.6	46.60	14.1	18.6	8.2	5.7	11.3	22.1
ACTH pg/ml	-	13.9	10.4	5.1	12.14	7.58	<10	1.4	3.85	13.2	31.6	9.3	15	5.8	30
ODS cortisol mcg/dL	2.82	2.1	3.69	39.9	0.67	12.3	8.62	3.25	34.01	4.4	2.5	6.5	5.4	8.5	1.95
DHEAS mcg/L	-	-	<15	29	915.4	-	-	73.8	28.2	34.2	144	69.2	121	-	16
Testosterone ng/ml	-	-	0.04	0.28	2.7	-	-	0.34	0.16	0.15	3.08	0.06	-	-	-
DRC uIU/mL	-	-	2.8	-	5.9	1.9	-	-	8.5	-	9.6	-	32	-	-
PRA ng/ml/h	-	0.55	-	-	-	-	-	-	-	1.57	-	-	-	0.22	-
PAC ng/dL	-	2.9	3.66	-	13.1	5.3	-	-	1.4	24	9.5	2.6	10.2	13.3	-
PFNMN pg/mL	81.7	-	-	-	7.15	-	-	-	-	-	60	51			-
PFMN pg/mL	54.7	-	-	-	9.45	-	-	-	-	-	-	-			-
Management	Observation	Observation	Observation	B/L Adrenalectomy	B/L Adrenalectomy	Observation	Observation	Observation	B/L Adrenalectomy	Observation	Observation	Observation	Observation	Awaiting B/L adrenalectomy	Observation
Follow up duration	11.9 years	12.4 years	11 years	-	5 years	1.5 years	-	4.8 years	8 months	-	6 years	-	6.7 years	-	-
Outcomes	Stable No new comorbidities	Stable No new comorbidities	Stable No new comorbidities	Cured	Cured	Stable No new comorbidities	Expired (accidental)	Stable Developed Diabetes Started on ketoconazole at follow-up	Cured	-	Stable No new comorbidities	-	Stable No new comorbidities	-	-
Genetics	NA	NA	Negative	Negative	Negative	Negative	NA	Negative	*ARMC5*	NA	NA	NA	*MEN1*	Awaited	NA

Abbreviations: ACTH – Adrenocorticotrophic hormone, CS – Cushing syndrome, DHEAS – Dehydroepiandrostenedione sulphate, DM – Diabetes Mellitus, DRC – Direct Renin Concentration, HTN – Hypertension, NA – Not available, ODS – overnight dexamethasone suppression, PAC – Plasma Aldosterone Concentration, PFNMN – Plasma free normetanephrine, PFMN – Plasma free metanephrine, PRA – Plasma Renin Activity

Supplementary Data

Supplementary Table 2 Non-secretory Group – Clinical, biochemical, genetics, treatment and outcome details

	Patient 16	Patient 17	Patient 18	Patient 19	Patient 20	Patient 21	Patient 22
Age yrs	62	50	50	83	39	46	38
Sex	M	F	M	F	M	F	F
Presentation<br>1-overt CS;2- hirsutism, 3- Incidental, 4-DM, 5- HTN	3	3	3	3	3	3	4
Family history	-ve	-ve	-ve	-ve	-ve	-ve	-ve
Basal cortisol mcg/dl	9.6	13.2	14.4	6.8	13.3	7	11.1
ACTH pg/ml	11.8	9.4	20.5	19	118	12.9	21.2
ODS cortisol mcg/dL	1.2	0.9	1.2	0.9	0.9	1.2	1.51
DHEAS mcg/L	-	265	-	-	-	-	10.7
Testosterone ng/ml	-	0.11	-	-	-	0.57	0.02
DRC uIU/mL	-	36.2	-	-	24.2	-	
PRA ng/ml/h	-	-	-	-	-	-	1.04
PAC ng/dL	-	-	-	-	6.4	-	6.4
PFNMN pg/mL	-	-	-	43	56	-	85
PFMN pg/mL	-	-	-	25	-	-	15
Management	Observation	Observation	Observation	Observation	Observation	Observation	Observation
Outcomes	Stable	Stable	Stable	Stable	Stable	Stable	Stable
Genetics	NA	NA	NA	NA	NA	NA	*MEN1*

Abbreviations: ACTH – Adrenocorticotrophic hormone, CS – Cushing syndrome, DHEAS – Dehydroepiandrostenedione sulphate, DM – Diabetes Mellitus, DRC – Direct Renin Concentration, HTN – Hypertension, NA – Not available, ODS – overnight dexamethasone suppression, PAC – Plasma Aldosterone Concentration, PFNMN – Plasma free normetanephrine, PFMN – Plasma free metanephrine, PRA – Plasma Renin Activity

Supplementary Table 3: LCMS/MS steroid profile of BMAD patients

Parameter	Patient 9	Patient 10	Patient 12	Ref. Range
Aldosterone (ng/L)	14.0	240	26.4	-
Androstenedione (ng/L)	370	560	358	-
Cortisol (ug/L)	226	79.2	82.5	-
Cortisone (ug/L)	18.1	18.3	13.2	6-27
Corticosterone (ug/L)	12.2	0.93	1.72	1-20
11-Deoxycortisol (ug/L)	0.68	0.61	0.36	0.5-3.0
21-Deoxycortisol (ug/L)	<0.03	<0.027	<0.03	0.02-0.15
DHEA (ug/L)	0.11	0.69	0.31	-
DHEAS (ug/L)	282	342	692	M: 99-6154<br>F: <5270
11- Deoxycorticosterone (ug/L)	0.21	0.03	0.05	0.02-0.15
17-α-Hydroxyprogesterone (ug/L)	0.52	0.19	0.11	0.20-2.20
Progesterone (ug/L)	0.07	0.01	0.01	M: 0.19-1<br>F: -
Testosterone (ug/L)	0.16	0.15	0.06	M: 2.5-10<br>F: 0.20-0.80

Abbreviations: DHEA – Dehydroepiandrostenedione, DHEAS - Dehydroepiandrostenedione sulphate, F – Female, M – Male

Supplementary Table 4: Comparison of characteristics of BMAD patients in our cohort with literature

Author	Year	Place	N	M:F	Mean Age	*ARCM5 +*	Syndromic	Overt Cushing's	MACS	Non-secretory	Treatment
Hofland et al[1]	2013	Netherlands	35	9:26	56.1	NA	NA	22 (63)	13 (37)	0 (0)	NA
Espiard et al[2]	2015	France	98	63:35	53	24/92 (26)	NA	42 (43)	46 (47)	10 (10)	Surgery: 46 (47)
Albiger et al[3]	2016	Italy	72	20:52	58.3	12/71 (17)	NA	33 (46)	20 (28)	19 (26)	NA
Osswald et al[4]	2019	Germany	34	13:21	NA	2/10 (20)	None	29 (85.2)	5 (14.8)	0 (0)	ULA: 25 (74)<br>BLA: 9 (26)
Zhang et al[5]	2020	China	46	26:20	52.1	NA	NA	28 (61)	18(39)	0 (0)	ULA: 21(46)<br>BLA: 0 (0)<br>UTB: 8 (17)
Wurth et al[6]	2021	USA	44	13:31	53	11/44 (25)	NA	NA	NA	NA	NA
Bouys et al[7]	2022	France	352	122:230	55	52/352 (15)	NA	95 (27)	207 (59)	50 (14)	ULA: 79 (22)<br>BLA: 34 (10)
Wang et al[8]	2023	China	124	66:58	50.2	NA	NA	68 (54.8)	56 (45.2)	0 (0)	ULA: 60 (48)<br>BLA: 0 (0)<br>UTB: 64 (52)
Araujo-Castro et al[9]	2023	Spain	32	13:19	54.2	NA	NA	1 (3)	31(97)	0 (0)	ULA: 2 (6)<br>BLA: 0 (0)
Current study	2024	India	22	6:16	48.2	1/6 (17)	2/21 (*MEN1*)	3 (14)	10 (45)	7 (32)	ULA: 0 (0)<br>BLA: 4 (19)

Abbreviations: MACS – Mild autonomous cortisol secretion, BLA – Bilateral adrenalectomy, ULA – Unilateral adrenalectomy, UTB – Unilateral adrenalectomy followed by bilateral adrenalectomy

Supplementary Data

References:

1. Hofland J, Hofland LJ, van Koetsveld PM, et al. ACTH-independent macronodular adrenocortical hyperplasia reveals prevalent aberrant in vivo and in vitroresponses to hormonal stimuli and coupling of arginine-vasopressin type 1a receptor to 11β-hydroxylase. *Orphanet Journal of Rare Diseases*. 2013;8(1):142. doi:10.1186/1750-1172-8-142

2. Espiard S, Drougat L, Libé R, et al. ARMC5 Mutations in a Large Cohort of Primary Macronodular Adrenal Hyperplasia: Clinical and Functional Consequences. *The Journal of Clinical Endocrinology & Metabolism*. 2015;100(6):E926-E935. doi:10.1210/jc.2014-4204

3. Albiger NM, Regazzo D, Rubin B, et al. A multicenter experience on the prevalence of ARMC5 mutations in patients with primary bilateral macronodular adrenal hyperplasia: from genetic characterization to clinical phenotype. *Endocrine*. 2017;55(3):959-968. doi:10.1007/s12020-016-0956-z

4. Osswald A, Quinkler M, Di Dalmazi G, et al. Long-Term Outcome of Primary Bilateral Macronodular Adrenocortical Hyperplasia After Unilateral Adrenalectomy. *The Journal of Clinical Endocrinology & Metabolism*. 2019;104(7):2985-2993. doi:10.1210/jc.2018-02204

5. Zhang Q, Xiao H, Zhao L, et al. Analysis of clinical and pathological features of primary bilateral macronodular adrenocortical hyperplasia compared with unilateral cortisol-secreting adrenal adenoma. *Ann Transl Med*. 2020;8(18):1173. doi:10.21037/atm-20-5963

6. Wurth R, Tirosh A, Kamilaris CDC, et al. Volumetric Modeling of Adrenal Gland Size in Primary Bilateral Macronodular Adrenocortical Hyperplasia. *Journal of the Endocrine Society*. 2021;5(1):bvaa162. doi:10.1210/jendso/bvaa162

7. Bouys L, Vaczlavik A, Jouinot A, et al. Identification of predictive criteria for pathogenic variants of primary bilateral macronodular adrenal hyperplasia (PBMAH) gene ARMC5 in 352 unselected patients. *European Journal of Endocrinology*. 2022;187(1):123-134. doi:10.1530/EJE-21-1032

8. Wang W, Lian P, Deng J, Li H, Zhang X. A 30-Year, Single-Center Experience of Unilateral Adrenalectomy for Primary Bilateral Macronodular Adrenal Hyperplasia. *Endocrine Practice*. 2022;28(7):690-695. doi:10.1016/j.eprac.2022.04.011

9. Araujo-Castro M, Reincke M. Primary bilateral macronodular adrenal hyperplasia: A series of 32 cases and literature review. *Endocrinología, Diabetes y Nutrición*. 2023;70(4):229-239. doi:10.1016/j.endinu.2022.12.010

Supplementary Table 5: CT characteristics of BMAD patients described in the literature

Author	Year	N	Right adrenal nodule (cm)<br>Max dimension	Basal Attenuation<br>(HU)	Contrast attenuation (HU)	Left adrenal nodule (cm)<br>Max dimension	Basal Attenuation<br>(HU)	Contrast attenuation (HU)	Secretory status
Hannah-Shmouni et al[1]	2018	1	2	20	-	1.2	10		Estrogen
Sanchis et al[2]	2018	1	3.2	<10	-	2.7	30	-	MACS
Zhang et al[3]	2020	1	3.0[a]	5	-	3.5[a]	5	-	MACS
Higashitani et al[4]	2020	1	2.0	11	-	4.5	14	-	MACS
Bourdeau et al[5]	2016	4	2.9<br>.8<br>.8<br>1	25<br>-<br>-<br>24	-<br>-<br>-<br>-	5.9<br>2.3<br>2.5<br>2	24<br><10<br>26<br>26	-<br>-<br>-<br>-	MACS<br>MACS<br>MACS<br>MACS
Zhang et al[6]	2020	46	0.5 – 3.5[b]	8-24[b]	22-66[b]	-	-	-	Overt CS – 28<br>MACS – 18

[a]Adrenal gland dimension, [b]Range inclusive of both adrenals

Abbreviations: CS – Cushing’s Syndrome, HU – Hounsfield Unit, MACS – Mild autonomous cortisol excess

Supplementary Data

References:

1. Hannah-Shmouni F, Moraitis AG, Romero VV, et al. Successful Treatment of Estrogen Excess in Primary Bilateral Macronodular Adrenocortical Hyperplasia with Leuprolide Acetate. *Horm Metab Res*. 2018;50(02):124-132. doi:10.1055/s-0043-122074

2. Juliá-Sanchis M de los L, Navarro-Téllez M del P, Falcones-Gracia KV, Ricart-Álvarez E, González-Bueno MV, Molina-Gasset R. Two cases of Cushing's syndrome due to primary bilateral macronodular adrenal hyperplasia secondary to aberrant adrenal expression of hormone receptors. *Clinical Biochemistry*. 2018;59:86-89. doi:10.1016/j.clinbiochem.2018.06.013

3. Zhang F, Lin X, Yu X. Primary macronodular adrenal hyperplasia (PMAH) can be generated by a new ARMC5 germline variant (c.52C>T (p.Gln18X)). *Endocrine Journal*. 2020;67(12):1179-1186. doi:10.1507/endocrj.EJ20-0163

4. Higashitani T, Karashima S, Aono D, et al. A case of renovascular hypertension with incidental primary bilateral macronodular adrenocortical hyperplasia. *Endocrinology, Diabetes & Metabolism Case Reports*. 2020;2020(1). doi:10.1530/EDM-19-0163

5. Bourdeau I, Oble S, Magne F, et al. ARMC5 mutations in a large French-Canadian family with cortisol-secreting β-adrenergic/vasopressin responsive bilateral macronodular adrenal hyperplasia. *European Journal of Endocrinology*. 2016;174(1):85-96. doi:10.1530/EJE-15-0642

6. Zhang Q, Xiao H, Zhao L, et al. Analysis of clinical and pathological features of primary bilateral macronodular adrenocortical hyperplasia compared with unilateral cortisol-secreting adrenal adenoma. *Ann Transl Med*. 2020;8(18):1173. doi:10.21037/atm-20-5963
